# Editorial: Muscle mass and function in COVID-19

**DOI:** 10.3389/fnut.2022.1012742

**Published:** 2022-11-15

**Authors:** Caterina Conte, Maurizio Muscaritoli

**Affiliations:** ^1^Department of Human Sciences and Promotion of the Quality of Life, San Raffaele Roma Open University, Rome, Italy; ^2^Department of Endocrinology, Nutrition and Metabolic Diseases, Istituto di Ricovero e Cura a Carattere Scientifico (IRCCS) MultiMedica, Milan, Italy; ^3^Department of Translational and Precision Medicine, Sapienza University of Rome, Rome, Italy

**Keywords:** muscle, COVID-19, sarcopenia, long-COVID syndrome, SARS-CoV-2

The aim of this Research Topic is to explore the role of sarcopenia, i.e., reduced muscle mass and function, in COVID-19. The articles included in the Research Topic cover a wide range of aspects pertaining to the relationship between sarcopenia and COVID-19, spanning from novel technologies to disentangle the mechanisms underpinning the detrimental effects of SARS-CoV-2 on skeletal muscle, such as *in vitro* 3D cell culture models as reviewed by Seixas et al., to specific metabolic pathways potentially involved, such as the kynurenine pathway of tryptophan catabolism (Takeshita and Yamamoto) or those triggered by COVID-19-induced hypoxia and inflammation (Di Girolamo et al.). More in detail, Takeshita and Yamamoto delve into the complex interactions among the angiotensin converting enzyme 2 (ACE-2), which is the receptor for SARS-CoV-2, the kynurenine pathway and skeletal muscle function, suggesting a potential role for tryptophan in the maintenance of human health. Additionally, Di Girolamo et al. review the effects of COVID-19-induced hypoxia on muscle mass, providing evidence that this could lead to muscle wasting and the so called “long COVID”. Consistently, in their Original Research Article De Blasio et al. report a high prevalence of malnutrition and *dynapenia* (the reduction in muscle strength) in a cohort of post-acute COVID-19 patients referred to a rehabilitation center after hospital discharge. Patients exhibited marked abnormalities of handgrip strength and body composition parameters as assessed by bioelectrical impedance analysis, which were more prevalent in those bedridden or malnourished and when fat free mass or skeletal mass was low. These data suggest a deep impact of COVID-19 on skeletal mass and function, as well as on nutritional status, and are in line with previous literature showing that COVID-19 patients often experience a clinically significant weight loss, at least in part due to loss of muscle mass (1, 2). Of note, weight loss is followed by weight regain that is characterized by an increase in abdominal adiposity (3). These unfavorable changes in body composition may dramatically impact health and functional status. On the other hand, pre-existing poor skeletal muscle health may impact clinical outcomes of COVID-19 patients. Pinto et al. systematically review the available evidence on the relationship between skeletal muscle characteristics and COVID-19, to examine the association between muscle status and disease severity. They show that low muscle quality and function, rather than muscle quantity/mass, is associated with COVID-19 severity, and suggest that muscle function be included as a clinical predictor in the assessment of COVID-19 patients. Furthermore, we show that the presence of myosteatosis (increased intramuscular fat, as measured by CT-derived skeletal muscle radiation attenuation) diagnosed during acute COVID-19 increases the risk of persistent dyspnoea and mobility problems 6 months after discharge by 3.2 and 3.7 times, respectively (De Lorenzo et al.). These data are in line with the growing body of evidence stressing the importance of muscle quality in determining health outcomes, as also acknowledged by international guidelines on sarcopenia (4, 5).

Overall, the articles included in the Research Topic suggest a bi-directional relationship between COVID-19 and skeletal muscle health: on the one hand, SARS-CoV-2 may directly and indirectly affect skeletal muscle, possibly leading to sarcopenia. On the other hand, pre-existing poor muscle quality has a negative impact on clinical outcomes of COVID-19 patients, both in the short and in the long term. These considerations prompt the need for easy-to-implement strategies to prevent the adverse effects of COVID-19 on skeletal muscle health, and to improve muscle mass and function in patients who have or are at risk of sarcopenia. In their Brief Research Report, Detopoulou et al. show how simple menu changes and addition of comfort foods may substantially boost the nutrient content of the hospital diet. In fact, the authors report that relatively minor changes in diet composition result in significant increases in total energy content, protein and carbohydrate, as well as in leucine, several vitamins including vitamin C and D, and minerals including magnesium and selenium.

In conclusion, the evidence presented in this Research Topic highlights the importance of preserving not only muscle mass, but—and possibly even more importantly—muscle quality in order to improve short- and long-term clinical outcomes of COVID-19 patients. We believe that muscle health is a key target to improve overall health, and future research should aim at better defining biomarkers of muscle quality and identifying the best nutritional, pharmacological and exercise strategies to maintain skeletal muscle health, in the setting of COVID-19 but also in the broad range of acute and chronic conditions associated with sarcopenia.

It is with great personal satisfaction that we can say that the launch of this Research Topic turned out to be a very successful initiative for the Journal.

## Author contributions

CC drafted the manuscript. MM revised it critically for important intellectual content. CC and MM provided approval for publication of the content and agreed to be accountable for all aspects of the work. All authors contributed to the conception of the work. All authors contributed to the article and approved the submitted version.

## Conflict of interest

The authors declare that the research was conducted in the absence of any commercial or financial relationships that could be construed as a potential conflict of interest.

## Publisher's note

All claims expressed in this article are solely those of the authors and do not necessarily represent those of their affiliated organizations, or those of the publisher, the editors and the reviewers. Any product that may be evaluated in this article, or claim that may be made by its manufacturer, is not guaranteed or endorsed by the publisher.
